# Attentional modulations of the early and later stages of the neural processing of visual completion

**DOI:** 10.1038/srep08346

**Published:** 2015-02-10

**Authors:** Xiang Wu, Liang Zhou, Cheng Qian, Lingyu Gan, Daren Zhang

**Affiliations:** 1 Department of Psychology, Sun Yat-Sen University, China; 2 CAS Key Laboratory of Brain Function and Disease, and School of Life Sciences, the University of Science and Technology of China, Hefei, Anhui, China

## Abstract

The brain effortlessly recognizes objects even when the visual information belonging to an object is widely separated, as well demonstrated by the Kanizsa-type illusory contours (ICs), in which a contour is perceived despite the fragments of the contour being separated by gaps. Such large-range visual completion has long been thought to be preattentive, whereas its dependence on top-down influences remains unclear. Here, we report separate modulations by spatial attention and task relevance on the neural activities in response to the ICs. IC-sensitive event-related potentials that were localized to the lateral occipital cortex were modulated by spatial attention at an early processing stage (130–166 ms after stimulus onset) and modulated by task relevance at a later processing stage (234–290 ms). These results not only demonstrate top-down attentional influences on the neural processing of ICs but also elucidate the characteristics of the attentional modulations that occur in different phases of IC processing.

The brain recognizes objects even when environmental information is widely separated. Such large-range visual completion is demonstrated by the Kanizsa-type illusory counters (ICs. The present study focused on Kanizsa-type ICs. Other types of ICs are discussed in the third paragraph of the Discussion section), in which a contour is perceived despite the widely separate contour edges^1^ (Fig. 1a).

Despite the effortless processing of ICs^2^,^3^,^4^,^5^ and the involvements of early visual areas (V1 and V2) in IC perception^6^,^7^,^8^, high-level cognitive processes have long been believed to be necessary for IC processing^9^,^10^,^11^. This cognitive view suggests that the perceived ICs are postulated based on object clues in the spatial configuration of the stimulus and Kanizsa-type ICs are thus regarded as “cognitive contours”. It is further suggested that IC processing involves two stages: an early “postulating stage” in which an object is postulated based on spatial stimulus configuration, and a later “matching stage” in which the analyzed object information is compared with the postulated object^11^. Accordingly, the lateral-occipital cortex (LOC) has been found to be involved in IC perception^12^,^13^,^14^. Specifically, event-related potential (ERP) studies with high temporal resolution have shown two LOC-localized ERP components that are sensitive to ICs: the negative difference waveforms between Kanizsa and control stimuli occurring at approximately 146 and 260 ms after stimulus onset, respectively^15^,^16^,^17^,^18^. The spatiotemporal characteristics of the early and later IC-sensitive ERP components allow them to be the neural candidates that represent the postulating and matching stages, respectively.

The essence of the cognitive view is that the top-down influences from high-level cognitive processes produce object postulations, and one testing approach is to examine the attentional modulation of IC perception. However, the neural evidence supporting the attentional influences on IC perception is surprisingly sparse, and the existing reports do not appear to support the attentional effects; e.g., the early IC-sensitive ERP component is not influenced by whether the ICs are attended (i.e., active vs. passive tasks)^16^,^19^. Attentional resources can be allocated according to either spatial or object information^20^,^21^. We propose that the postulating stage may involve more spatial attention (e.g., the size of the attentional window) because the external spatial stimulus configuration is required to cue the object, whereas the matching stage may be more sensitive to task relevance (i.e., whether the ICs are attended) because the processed object information is compared with the internally represented object postulation. Such a two-stage attentional mechanism would also account for the reported invulnerability of the early IC-sensitive ERP component to task relevance because it may be more sensitive to spatial attention than to task relevance.

The proposed two-stage attentional mechanism of IC perception was examined in the present study by manipulating spatial attention and task relevance independently and recording the early and later IC-sensitive ERP components.

## Results

**Figure 1:**
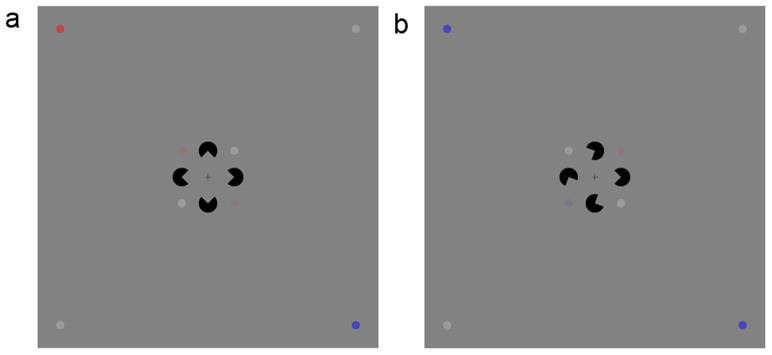
Illustration of experimental stimuli. Two sample stimuli are shown. Each stimulus consisted of a Kanizsa (a) or a control (b) figure, four central points with two colored points in either the same (a) or different (b) colors, and four peripheral points with two colored points in either the same (b) or different (a) colors.

The experimental stimuli contained either a Kanizsa or a control figure, four central points, and four peripheral points (Fig. 1). The subjects were asked to indicate whether a diamond appeared, whether the two central color points were the same color, or whether the two peripheral color points were the same color. The three tasks were henceforth referred as the Kanizsa figure (KF), central color (CC), and peripheral color (PC) tasks, respectively. The KF task was task relevant (i.e., related to the IC shape), whereas the CC and PC tasks were task irrelevant. The KF and CC tasks had a small spatial attentional window with the four central points at the corners of the window, whereas the PC task had a large spatial attentional window with the four peripheral points at the corners of the window.

**Figure 2:**
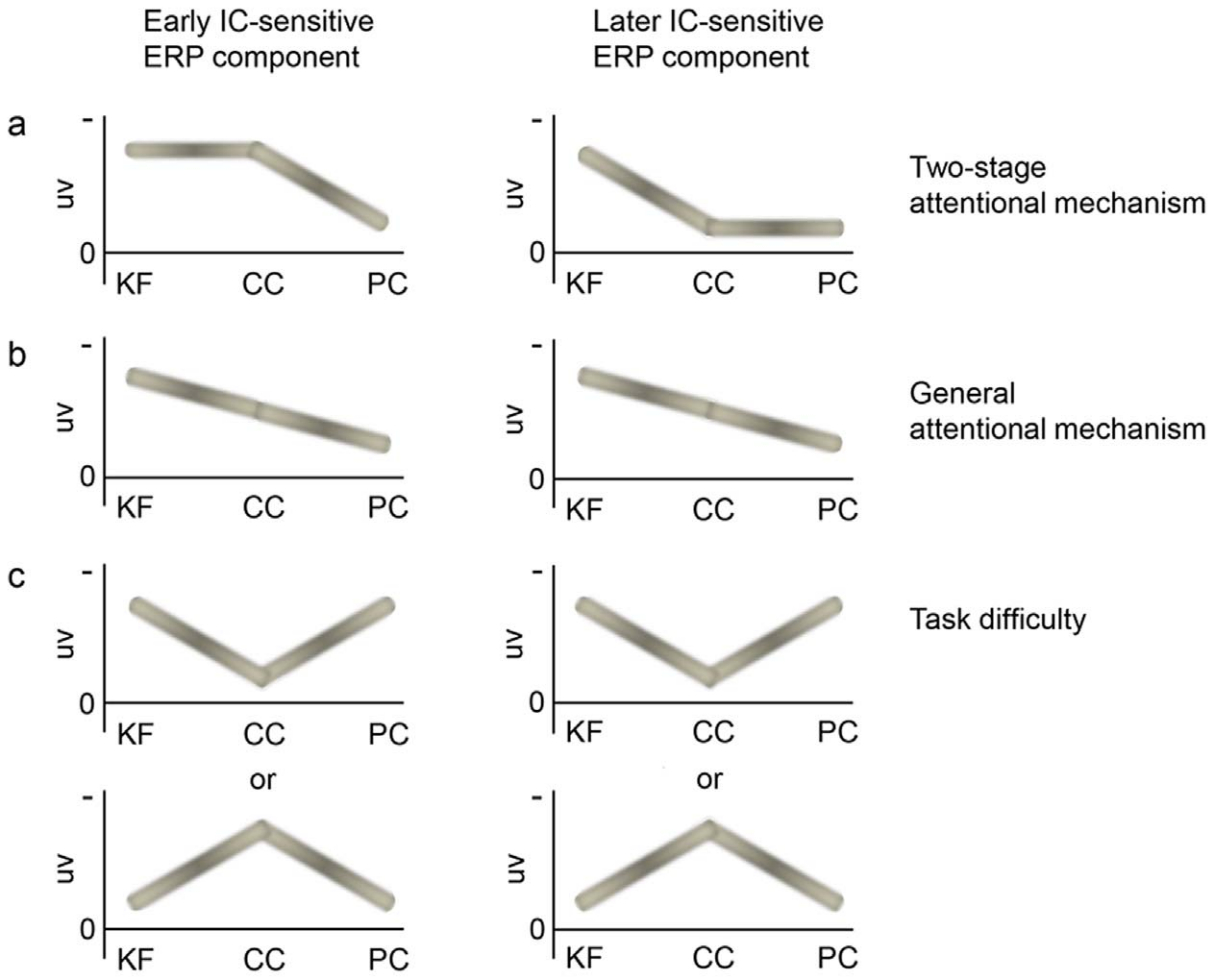
Diagram of the predictions regarding the neural processing of illusory contours (IC) based on three possible mechanisms. (a): The two-stage attentional mechanism predicts that the early IC-sensitive ERP component would not differ between the KF and CC tasks and would be weaker in the PC task, and that the later IC-sensitive ERP component would be weaker in the CC and PC tasks and would not differ in the two tasks. (b): The general attentional mechanism predicts that both the early and later IC-sensitive ERP components would be weaker in the CC than in the KF task and weaker in the PC than in the CC task. (c): Task difficulty (see Fig. 3) predicts that both the early and later IC-sensitive ERP components would be weaker in the CC than in the KF task and stronger in the PC than in the CC task (or stronger in the CC than in the KF task and weaker in the PC than in the CC task). KF, CC, and PC indicate the three tasks in which the subjects judged whether a Kanizsa figure appeared, whether the two central color points were the same color, or whether the two peripheral color points were the same color, respectively. The y-axis indicates the amplitude (in uv) of the ERP components. Here we plot negative (indicated by the negative sign) amplitude up, because the early and later IC-sensitive ERP components are supposed to be negative and a larger negativity appears to indicate a larger magnitude response related to contour processing. (Note that the diagrams in Fig. 2 are drawn to help illustrate the logic behind the present design. We emphasize that the diagrams reflect comparisons between tasks rather than specific patterns across tasks. For instance, the general attentional mechanism predicts that IC-sensitive ERP components would be stronger in the KF than in the CC task and stronger in the CC than in the PC task, as illustrated in Fig. 2b. However, we do not intend to indicate a strict linear change in the IC-sensitive ERP components across the KF, CC, and PC tasks).

**Figure 3:**
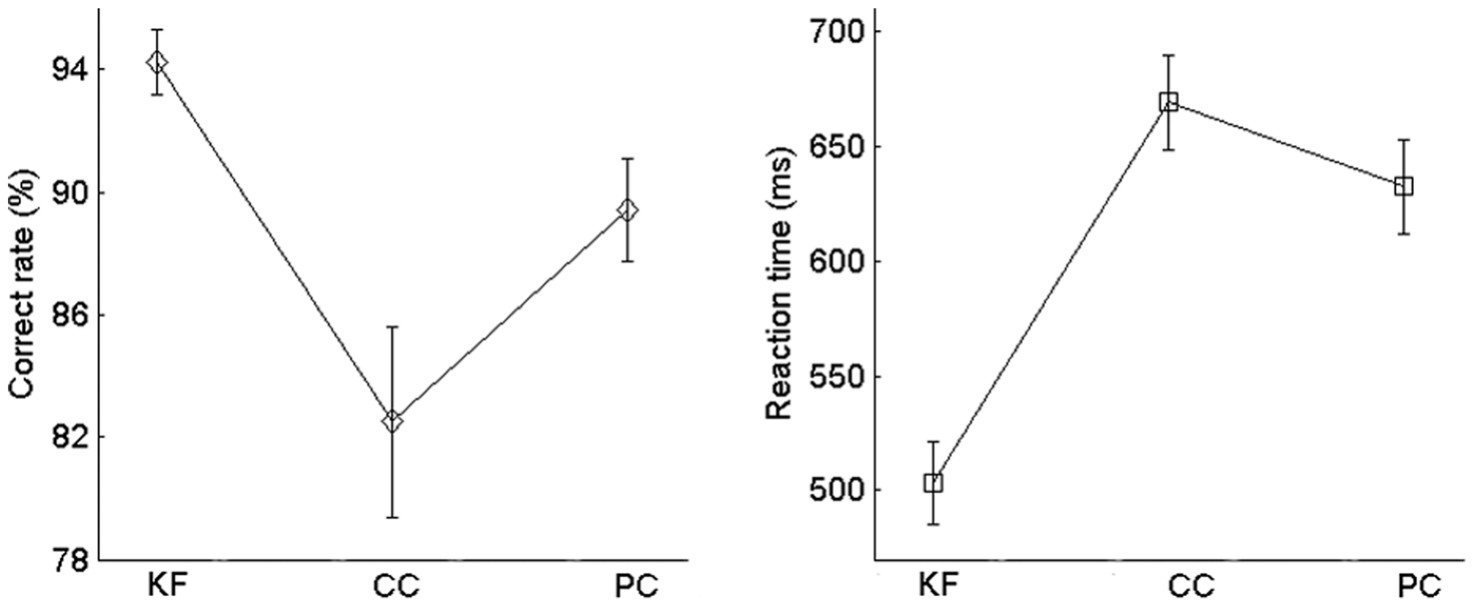
Behavioral results. The CC task was the most difficult. The error bars indicate ± S.E.M.

The current design also allowed us to differentiate the effects predicted by the proposed two-stage attentional mechanism from those predicted by a general attentional mechanism or task difficulty. The two-stage attentional mechanism predicts that the early IC-sensitive ERP component should not differ between the KF and CC tasks and should be weaker in the PC task, whereas the later IC-sensitive ERP component should be weaker in the CC and PC tasks and should not differ between the two tasks (Fig. 2a). By contrast, the hypothesis of a general attentional mechanism (i.e., the effects of spatial attention and task relevance do not differ across processing stages) would predict that both the early and later IC-sensitive ERP components should be weaker in the CC than in the KF task and weaker in the PC than in the CC task (Fig. 2b) because, compared with the KF task, attention would be drawn away from the ICs in the CC task and further spatially drawn away from the region of the ICs in the PC task. Task difficulty may also potentially influence the neural responses. In our preliminary testing (in which the central and peripheral colored points were equally visible) of the experimental design, we observed that the difficulties of the three tasks were KF < CC < PC. This variation in task difficulty in the three tasks could influence the neural results similarly to the general attention effect (given that it is unclear whether task difficulty would enhance or reduce IC processing). Therefore, the central colored points were made less visible than the peripheral colored points in the formal experiment (Fig. 1) such that the CC task was the hardest (see Fig. 3 below for behavioral results). Therefore, in the current design, if task difficulty influenced neural results, both the early and later IC-sensitive ERP components should be weaker in the CC than in the KF task and stronger in the PC than in the CC task (or stronger in the CC than in the KF task and weaker in the PC than in the CC task) (Fig. 2c).

The behavioral results are depicted in Fig. 3. A one-way ANOVA with the factor task (three tasks) was conducted for both the correct rate and reaction time. A significant main effect for the factor task was observed for both the correct rate (*F*_2,26_ = 12.12, *p* = 0.003, partial η^2^ = 0.48) and the reaction time (*F*_2,26_ = 183.12, *p* < 0.001, partial η^2^ = 0.93). Post-hoc analyses showed that the correct rate was higher in the KF task than in the CC (*t*_13_ = 4.14, *p* = 0.001, η^2^ = 0.57) and PC (*t*_13_ = 5.90, *p* < 0.001, η^2^ = 0.73) tasks, and was lower in the CC than in the PC task (*t*_13_ = 2.42, *p* = 0.031, η^2^ = 0.31). Reaction time was faster in the KF task than in the CC (t_13_ = 15.85, *p* < 0.001, η^2^ = 0.95) and PC (t_13_ = 14.10, *p* < 0.001, η^2^ = 0.94) tasks, and was slower in the CC than in the PC task (t_13_ = 5.15, *p* < 0.001, η^2^ = 0.67).

**Figure 4:**
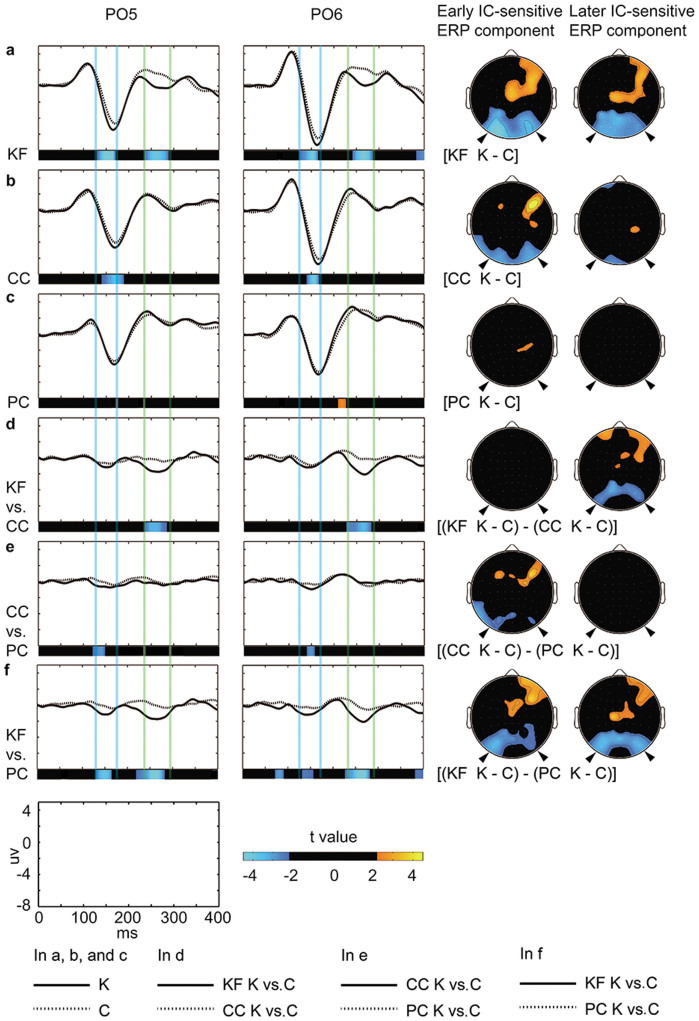
ERP results. (a–c): Left: ERP waveforms in the three tasks. The ERP waveforms elicited by the Kanizsa and control stimuli are drawn with solid and dashed lines, respectively. Below the waveform images are the statistical maps that show the time periods in which the two waveforms were significantly different (black indicates non-significance differences) according to the contrasts [KF K minus C], [CC K minus C], and [PC K minus C]. K refers to the Kanizsa figure and C refers to the control figure. The time period of the early IC-sensitive ERP component (130–166 ms) is marked by the two transparent blue vertical lines, and the time period of the later IC-sensitive ERP component (234–290 ms) is marked by the two transparent green vertical lines (the time periods were defined according to the KF task). (see methods below for more details). The representative electrodes PO5 and PO6 are shown. Right: The corresponding statistical topographic maps. The white points indicate the electrodes. The arrows indicate the scalp areas where the IC-sensitive ERP components occurred. (d–f): Left: ERP difference waveforms (between the Kanizsa and control stimuli) in the three direct comparisons between tasks. The difference waveforms for the former and the later components in each comparison are drawn with solid and dashed lines, respectively. Below the waveform images are the statistical maps that show the time periods in which the two waveforms were significantly different according to the contrasts [(KF K minus C) minus (CC K minus C)], [(CC K minus C) minus (PC K minus C)], and [(KF K minus C) minus (PC K minus C)]. Right: The corresponding statistical topographic maps. The axes for the waveform images, the color bar for the statistical maps and statistical topographic maps, and the legends of the solid and dashed lines for ERP waveforms (a–c) and ERP difference waveforms (d–f) are shown at the bottom.

The ERP results are depicted in Fig. 4. The ERPs elicited by the Kanizsa figure (K) were compared to the ERPs elicited by the control figure (C) using the contrast [K minus C] for each of the three tasks separately. The time periods of the early and later negative IC-sensitive ERP components were defined according to the comparison results of the KF task (see methods below for more details). In the KF task, both the early (130–166 ms after stimulus onset) and later (234–290 ms) IC-sensitive ERP components were observed at the bilateral occipito-parietal electrodes (Fig. 4a). These results are consistent with previously reported early and later IC-sensitive ERP components^15^,^16^,^17^,^18^,^19^,^22^,^23^. The early IC-sensitive ERP component was also observed in the CC task (Fig. 4b), but not in the PC task (Fig. 4c). The later IC-sensitive ERP component was not observed in the CC or PC tasks. These attentional modulations were further confirmed through the direct comparisons between tasks (i.e., the differences in the ERP difference waveforms [K minus C] between the three tasks). For the early IC-sensitive ERP component, the contrast [(KF K minus C) minus (CC K minus C)] (Fig. 4d) showed no significant difference, whereas the contrasts [(CC K minus C) minus (PC K minus C)] (Fig. 4e) and [(KF K minus C) minus (PC K minus C)] (Fig. 4f) showed significant differences. For the later IC-sensitive ERP component, the contrasts [(KF K minus C) minus (CC K minus C)] and [(KF K minus C) minus (PC K minus C)] showed significant differences, whereas the contrast [(CC K minus C) minus (PC K minus C)] showed no significant difference. These results are in accordance with the predictions of the two-stage attentional mechanism of IC perception.

The IC-sensitive ERP components were further examined using a two-way ANOVA with the factors task (three tasks) and hemisphere (bilateral occipito-parietal electrodes PO5 and PO6). The time periods of the early and later IC-sensitive ERP components were used (a leave-one-subject-out (LOSO) approach was adopted to avoid non-independence in data analysis^24^). A significant main effect for the factor task was found for both the early (*F*_2,26_ = 6.01, *p* = 0.009, partial η^2^ = 0.32) and later (*F*_2,26_ = 16.89, *p* < 0.001, partial η^2^ = 0.57) IC-sensitive ERP components. There was no significant main effect for the factor hemisphere and no significant interaction between the factors task and hemisphere; thus, the data from two hemispheres were combined in post-hoc analyses. The early IC-sensitive ERP component was not significantly different between the KF and CC tasks, and was larger in the KF than in the PC task (t_13_ = 3.24, *p* = 0.006, η^2^ = 0.45) and larger in the CC than in the PC task (t_13_ = 2.50, *p* = 0.027, η^2^ = 0.32). The later IC-sensitive ERP component was larger in the KF task than in the CC (t_13_ = 3.92, *p* = 0.002, η^2^ = 0.54) and PC (t_13_ = 7.24, *p* < 0.001, η^2^ = 0.80) tasks, and was not significantly different between the CC and PC tasks. These results are consistent with the observations from the statistical maps and statistical topographic maps. Furthermore, the results of the ANOVA analyses performed on more occipito-parietal electrodes (left electrodes: PO3, PO5 and PO7; and right electrodes: PO4, PO6, and PO8) were consistent with the results of the above ANOVA analyses performed on the representative electrodes PO5 and PO6, showing significant main effects of the factor task for both the early (*F*_2,26_ = 5.62, *p* = 0.012, partial η^2^ = 0.30) and later (*F*_2,26_ = 15.11, *p* < 0.001, partial η^2^ = 0.54) IC-sensitive ERP components. These electrodes of interest were selected according to the well-characterized topographies for the early and later IC-sensitive ERP components as shown in Fig. 4 and previous studies^16^,^17^,^18^.

**Figure 5:**
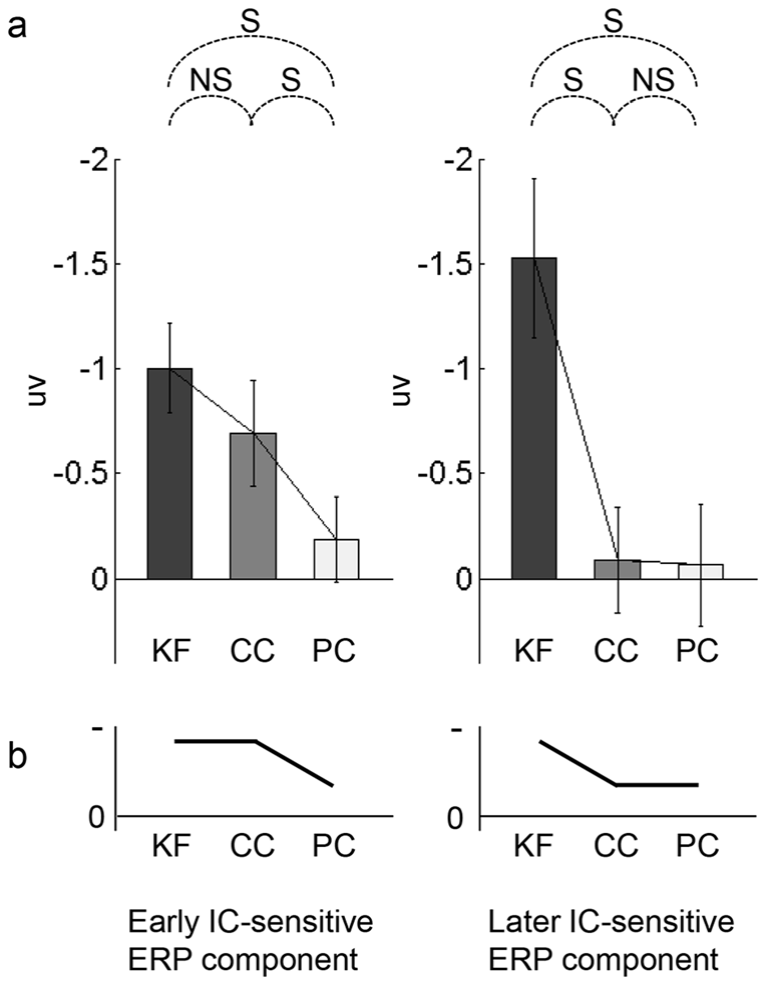
Differences in IC-sensitive ERP components between tasks. (a): The mean values (in uv) of the early and later IC-sensitive ERP components in the different tasks. The data are from the representative electrodes PO5 and PO6. The error bars indicate ± S.E.M. S indicates a significant comparison and NS indicates a non-significant comparison. (For a convenient comparison between the data and the predictions illustrated in Fig. 2, the negative amplitude is plotted up). (b): Diagram of the effects of the comparisons between tasks according to the significances of those comparisons. The conventions are as in Fig. 2.

The differences in IC-sensitive ERP components between tasks are summarized in Fig. 5. As clearly depicted in Fig. 5b, the current data are unlikely to support the predictions of the general attentional mechanism (Fig. 2b) or task difficulty (Fig. 2c), but agree with the predictions of the two-stage attentional mechanism (Fig. 2a). (The early IC-sensitive ERP component appeared to be stronger in the KF than in the CC task, which may indicate that the early IC-sensitive ERP component was also modulated by the general attentional mechanism. However, this was unlikely to be a robust effect (t_13_ = 1.22, *p* = 0.244, η^2^ = 0.10, in the test on PO5 and PO6; and t_13_ = 1.25, *p* = 0.233, η^2^ = 0.11, in the test on PO3, PO5, PO7, PO4, PO6 and PO8. See also the corresponding statistical map and statistical topographic map in Fig. 4 d)).

Next, we examined whether the results of the source reconstruction were consistent with the above results. We conducted two types of source analyses; the first involved source estimates of the group average difference waveforms (with high signal-to-noise ratios but without statistical information), and in the second source analysis source estimates of the difference waveforms were obtained for individual subjects and were then subjected to group statistical analyses (the signal-to-noise ratios were low, but statistical information was provided). The purpose was to examine the consistency between the two types of source analyses. Source estimates for the two time windows of interest (130–166 ms and 234–290 ms; i.e., the time periods of the early and later IC-sensitive ERP components) were generated.

**Figure 6:**
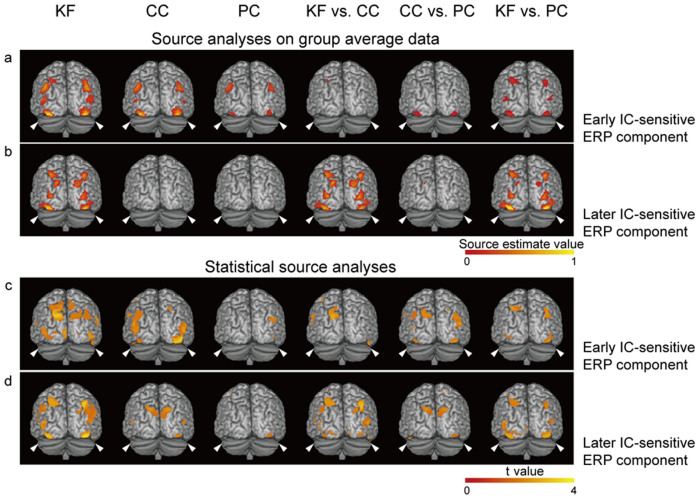
Source reconstruction results. (a): Source estimates of the group average ERP difference waveforms (corresponding to the contrasts [KF K minus C], [CC K minus C], [PC K minus C], [(KF K minus C) minus (CC K minus C)], [(CC K minus C) minus (PC K minus C)], and [(KF K minus C) minus (PC K minus C)]) for the time window of the early IC-sensitive ERP component. The arrows indicate the strongest source activities underlying the early IC-sensitive ERP component. Source estimate values were scaled to 0–1 for presentation. The source estimate values above 0.1 are shown. (b): For the time window of the later IC-sensitive ERP component. The conventions are as in (a). (c, d): The statistical maps from the statistical source analyses (uncorrected *p* < 0.05). The conventions are as in (a and b). The source activities are presented on a template rendered brain.

In the source analyses on the group average difference waveforms, both the early and later IC-sensitive ERP components were localized to the bilateral lateral occipital and posterior parietal cortices, with the strongest source activities in the lateral occipital regions (maximal coordinates in MNI space: left −38 −80 −16; and right 38 −82 −16). Consistent with the above analyses, for the time window of the early IC-sensitive ERP component, strong lateral occipital activities were observed in the KF and CC tasks, but not in the PC task (Fig. 6a). For the time window of the later IC-sensitive ERP component, strong lateral occipital activities were observed only in the KF task (Fig. 6b). The statistical source analyses (Fig. 6 c and d) provided results that were congruent with the results of the source analyses on the group average difference waveforms. These source reconstruction results are in accordance with the results of previous source reconstruction^16^,^17^,^18^,^19^,^25^ and functional magnetic resonance imaging (fMRI)^12^,^13^,^14^,^15^,^16^ studies that have investigated IC perception, and further revealed the attentional modulations of IC-sensitive source activities during different processing stages. It should be pointed out that the current data did not provide the spatial resolution necessary to precisely delimit the retinotopic borders of the observed lateral occipital source activities. Contributions from V4/V3a, which are adjacent to the LOC, were possible^12^, but V1 and V2 were highly unlikely to make a contribution to the observed source activities. (Note that, due to the low signal-to-noise ratios of difference waveforms of the individual subjects, the statistical powers in the statistical source analyses for the difference waveforms (Fig. 6 c and d) were not as strong as the statistical powers in the statistical source analyses for the original ERP waveforms, e.g., P1 and N1 (see Fig. S1). Therefore, the maps in Fig. 6 c and d are presented with a low statistical criterion (uncorrected *p* value < 0.05). However, importantly, the locations of the sources of the IC-sensitive ERP components and the attentional modulations of the source strengths in the different tasks were highly consistent between the statistical source analyses and the source analyses on the group average difference waveforms).

In the current study, we focused on the differences between the ERPs elicited by the Kanizsa and control figures that reflect the brain activities related to the perception of illusory contours. As shown in Fig. 4 a, b, and c, for both the Kanizsa and control figures, the typical visual evoked potential (VEP) components including P1 and N1 are clearly observed. Further analyses of the P1 and N1 in the KF task are presented in Fig. S1, to further support the validity of the present ERP data.

## Discussion

To examine the top-down attentional influences on the neural processing of large-range visual completion, the present study investigated the modulations of the ERP components that are sensitive to the perception of Kanizsa-type illusory contours by spatial attention and task relevance. We found that the early and later IC-sensitive ERP components were modulated by spatial attention and task relevance, respectively. The results also revealed that these attentional modulations of IC processing were unlikely to be due to the general attentional mechanism or task difficulty.

The results strongly suggest the presence of top-down attentional influences on IC processing and are thus consistent with the cognitive view that high-level cognitive processes are necessary for IC perception^9^,^10^,^11^. The results further suggest that spatial attention and task relevance affect the early and later stages of IC processing, respectively. Such a two-stage attentional mechanism is consistent with the characteristics of the early (i.e., an object postulating stage that requires the spatial stimulus configuration) and later (i.e., an object matching stage that requires the internal representation of the postulated objects) stages of IC processing proposed by the cognitive view^11^. A two-stage object closure (the closing of the fragments of objects) mechanism (early perceptual and later conceptual processes)^26^,^27^ has been adopted to account for the early and later IC-sensitive ERP components^17^,^18^,^19^,^23^. The present attentional model and the closure model may not conflict. Both models suggest that there are two stages of object-related processing in IC perception, and the attentional model emphasizes how attention is involved in the two stages of IC processing.

Because the early and later IC-sensitive ERP components reflect object-related processing in the LOC^15^,^16^,^18^,^19^,^25^,^28^, a reasonable prediction based on the current findings would be that, if the LOC activity is modulated by attention, the activities of the lower-tier visual areas may also be modulated by attention due to feed-back signals from the LOC to the lower-tier visual areas^13^,^16^. Perhaps partially due to the limitations of the spatial resolution of ERPs, clear evidence for IC-sensitive ERP components localized to the lower-tier visual areas is currently lacking. Thus, this prediction could be more appropriately examined with fMRI, which has finer spatial resolution. However, the prediction appears to contradict the results from an fMRI study that showed that the activations of the lower-tier visual areas (V1, V2, V3, and V4) elicited by illusory contours in neon color spreading figures are not modulated by attention (attention was controlled by judging the orientation of a bar above the fixation point)^29^. We believe that the results of the study of Sasaki and Watanabe^29^ do not warrant the conclusion that the activities of the lower-tier visual areas elicited by illusory contours are not modulated by attention, at least for Kanizsa-type illusory contours. The illusory contours of Kanizsa figures and the illusory contours of neon color spreading figures may involve different neural mechanisms. For example, the surface that is surrounded by the illusory contours of neon color spreading figures has been shown to be processed in V1^29^, whereas this processing occurs in the LOC for Kanizsa figures^13^. Moreover, Kanizsa figures are modal figures in which the completed contours are not occluded (and in which brightness is concurrently enhanced). Illusory contours can also be observed in amodal figures in which the completed contours are occluded (without concurrent brightness enhancement)^7^,^17^,^30^, and the processing of modal and amodal figures may have different neural substrates^3^,^7^,^17^. Furthermore, illusory contours can be defined by displaced gratings^31^,^32^,^33^, and Kanizsa-type and displaced-grating illusory contours may engage different neural mechanisms^12^,^31^,^32^. Certainly, different types of illusory contours might have shared neural substrates and are sometimes not strictly distinguished in the literature^29^,^33^,^34^. However, as mentioned above, converging evidence indicates that the characteristics of each type of illusory contour should also be taken into account. The conclusions drawn from the current results should be limited to Kanizsa-type illusory contours and would be further examined in studies comparing different types of illusory contours.

In Kanizsa-type illusory contour figures, a surface is surrounded by a contour. One question is whether the surface and the contour are supported by the same neural substrates. An fMRI study found similar LOC activities in response to a figure in which the surface is surrounded by a clear contour and a corresponding figure without a clear contour^13^. The authors of this study suggested that the LOC activity reflects a fast but crude surface-based processing that subsequently guides later detailed processing in the low-tier visual areas. This interpretation of the LOC activity is similar to the concept of early object postulation processing in the LOC, as reflected by the early IC-sensitive ERP component in the current study, which focused on the temporal stages of IC processing. Because the ERP data lack the spatial resolution necessary to differentiate the activities elicited by the surface and the contour, we emphasize that the current findings are discussed in terms of the perception of Kanizsa-type IC figures in general and not with respect specifically to surfaces or contours. The study by Sasaki and Watanabe^29^ is an elegant example of the use of fMRI retinotopic mapping methods for the isolation of activities elicited by surfaces from those elicited by the contours in neon color spreading figures. Future studies employing fMRI retinotopic mapping methods would allow for further clarification of the attentional modulations of neural responses to surfaces and contours of Kanizsa-type IC figures. Furthermore, the LOC has been shown to contain sub-regions (i.e., posterior and anterior regions)^35^,^36^, and the multivariate (pattern-based) approach is capable of identifying a greater amount of information from fMRI data than the conventional univariate (voxel-based) approach (for review, see Ref. 37). Whether different LOC sub-regions (or different activation patterns in the LOC) are related to the perception of surfaces and contours, respectively, and how the surface- or contour-related LOC activities are modulated by attention, remain open questions.

Whether the early stage of IC processing is automatic is a matter of intense debate^3^,^11^. An automatic process indicates that the process does not require attention or/and consciousness. The current study showed that the early stage of IC processing as reflected by the early IC-sensitive ERP component was modulated by spatial attention. It is possible that the early stage of IC processing does not require consciousness. This is also related to how the information processed at the early stage is utilized at a later stage. The early IC-sensitive ERP component may reflect unconsciously automatic contour processing and the later IC-sensitive ERP component may reflect a decision stage in which the stimulus information is used to determine if and how to respond^38^. The current study was not designed to investigate the modulation of IC processing by consciousness and the relationship between attention and consciousness per se is an open question^39^,^40^. These issues remain to be addressed in future studies that directly examine the influence of consciousness on the neural processing of ICs.

In summary, the vivid perception of Kanizsa-type illusory contours reveals the brain's ability to complete widely separated visual information. The present study demonstrated that spatial attention and task relevance modulate the neural activities elicited by ICs at the early and later stages, respectively. These findings are in accordance with the cognitive view that high-level cognitive processes are necessary for the perception of ICs^9^,^10^,^11^.

## Methods

### Participants

Fourteen right-handed subjects from the University of Science and Technology of China (nine males, mean age ± SD 23.4 ± 2.3 years) participated in this study. A typical ERP study investigating Kanizsa-type illusory contours involves approximately fourteen subjects^28^, which was the number of subjects in the present study. All subjects had normal or corrected-to-normal vision. The research protocols of this study were approved by Sun Yat-Sen University and the University of Science and Technology of China. All subjects provided written informed consents. The methods were carried out in accordance with the approved guidelines.

### Stimuli and procedure

The stimuli contained either a Kanizsa or a control figure (which consisted of four inducers), four central points, and four peripheral points (Fig. 1). The four black (RGB: 0 0 0) inducers were arranged so that an illusory diamond appeared in the Kanizsa figure and disappeared in the control figure. The control figure was produced by randomly rotating three of the four inducers of the Kanizsa figure clockwise or counterclockwise by 35 or 65 degrees. The location of the fourth non-rotated inducer varied randomly. Therefore, the subjects could not adopt a spatial strategy to perceive the difference between the Kanizsa and control figures^19^. The support ratio (i.e., the ratio of the physically specified edge length to the total edge length of the contour) was 0.5. For the four central or peripheral points, two points on one diagonal were white (RGB: 155 155 155) and two points on the other diagonal were colored (red and blue; in either the same or different colors). The RGB values of the central colored points were 139, 121, and 121 for red and 121, 121, and 139 for blue; and the RGB values of the peripheral colored points were 190, 70, and 70 for red and 70, 70, and 190 for blue.

There were three experimental tasks in which spatial attention and task relevance were independently modulated. In task one, the subjects were asked to indicate whether they saw a diamond. In task two, the subjects were asked to indicate whether the two central colored points were the same color. In task three, the subjects were asked to indicate whether the two peripheral colored points were the same color. The three tasks were henceforth referred as the Kanizsa figure (KF), central color (CC), and peripheral color (PC) tasks, respectively. In all tasks, the subjects responded by pressing one of two keys on a computer keyboard.

The stimuli were displayed on a gray (RGB: 130 130 130) background with a black fixation cross (0.28° visual angle) permanently displayed at the center of the screen. The diameter of the inducer subtended 0.5° of visual angle, and the side length of the diamond subtended a visual angle of 2°, which is suggested to be an optimal size for investigating neural responses to ICs^41^. The diameter of the central or peripheral point subtended 0.48° of visual angle. The distances from the center of a central or peripheral point to the center of the fixation cross subtended visual angles of 4° or 23.18°, respectively. The stimuli were presented for 150 ms in a random order with inter-stimulus intervals that ranged between 900–1200 ms. There were 400 trials in each task and the sequence of the stimuli was the same for the three tasks. The numbers of the stimuli with Kanizsa or control figures, with same or differently colored central points, and with same or differently colored peripheral points, were equal. The sequence of the three tasks was counterbalanced across subjects. The assignment of responding hands was counterbalanced across subjects.

The present stimuli were spatially symmetric around the fixation point, and the subjects did not need to move their eyes to perform the tasks. Furthermore, the stimuli were presented for only 150 ms and thus voluntary eye movements were unlikely^8^. Therefore, eye movements were unlikely to have had a major influence on the current results.

### EEG data recording and analyses

#### EEG recording

The EEG was recorded using a Neuroscan (www.neuroscan.com) system with 64 Ag/AgCl electrodes referenced to the unlinked bilateral mastoids. The electrodes were placed according to the international 10–20 system and the impedance of each electrode was kept below 5 kΩ. Horizontal and vertical electrooculograms (HEOG and VEOG) were also recorded to monitor eye movements. The electrode positions were recorded using Polhemus Fastrak (http://www.polhemus.com). The data were sampled at 500 Hz and filtered with a 0.05–100 Hz band-pass filter.

#### ERP analyses

The EEG was analyzed using a customized toolbox (mfeeg. http://sourceforge.net/p/mfeeg) programmed with MATLAB (The Mathworks, Natick, MA, USA). The EEG was cut into epochs (100 ms before stimulus onset to 400 ms after stimulus onset; this epoch of interest was padded with 300 ms on both sides to avoid artifacts due to filtering). Epochs with HEOG/VEOG exceeding ±50 μv were excluded from further analyses. On average, 149.7 ± 47.4, 149.9 ± 48, 154.4 ± 53.4, 153.5 ± 52.7, 151.4 ± 51.7, and 154 ± 51.4 artifact-free epochs were obtained for the Kanizsa and control stimuli in the KF, CC, and PC tasks, respectively. There was no significant difference in the numbers of trials across the different conditions. The epochs were then re-referenced to the average reference and 0.5–30 Hz band-pass filtered. For each epoch, the mean baseline (−100 to 0 ms) value was removed from the entire epoch. Next, the epochs were averaged to obtain the ERPs to the Kanizsa and control stimuli in the KF, CC, and PC tasks, respectively.

#### Statistical methods

First, statistical maps of the ERPs were generated by point-wise t-tests and a significance criterion of p < 0.05 for at least 10 consecutive data points in time (20 ms) was used^16^,^42^. The statistical maps were generated for the following contrasts: [KF K minus C], [CC K minus C], and [PC K minus C]; i.e., the differences in the ERPs between the Kanizsa and controls stimuli in the three tasks. K refers to the Kanizsa figure and C refers to the control figure. The following contrasts were also applied: [(KF K minus C) minus (CC K minus C)], [(CC K minus C) minus (PC K minus C)], and [(KF K minus C) minus (PC K minus C)]; i.e., the differences in the ERP difference waveforms (K minus C) between the different tasks. Furthermore, t values instead of ERP values (uv)^43^ were used to draw the topographic maps, which resulted in the statistical topographic maps. A significance criterion of p < 0.05 for a topographic region subtending at least two electrodes was used. Second, activities of interest (the time periods were defined based on the statistical maps, and the electrodes were selected according to the statistical topographic maps) were subjected to the repeated measures analysis of variance (ANOVA) (Greenhouse-Geisser corrections were applied). Data from the electrodes of interest in the left and right hemispheres were averaged, respectively, in the ANOVA analyses. The second step of ANOVA analysis supplemented the first step of statistical mapping analysis with hemispheric effects and a more intuitive way to inspect the findings (e.g., Fig. 5). A leave-one-subject-out (LOSO) approach was applied to the definitions of time periods of interest in the ANOVAs to avoid non-independence in data analysis^24^.

#### Source localization

The brain sources underlying the ERP difference waveforms corresponding to the six contrasts mentioned above were also estimated. To the best of our knowledge, previous source reconstruction studies that have investigated Kanizsa-type IC perception^16^,^17^,^18^,^19^ have provided visualization rather than statistical analyses of the likely underlying sources of IC-sensitive ERP components. One primary reason for this approach may be to maintain the highest possible signal-to-noise ratio because of the low signal-to-noise ratios of the difference waveforms of individual subjects^16^. In the current study, we conducted two types of source analyses. The first involved source estimates of the group average difference waveforms (with high signal-to-noise ratios but without statistical information). In the second source analysis, source estimates of the difference waveforms were obtained for individual subjects and were then subjected to group statistical analyses (the signal-to-noise ratios were low, but statistical information was provided). The purpose was to examine the consistency between the two types of source analyses. The sources were analyzed using source reconstruction procedures implemented in SPM8^44^ (Wellcome Department of Cognitive Neurology. www.fil.ion.ucl.ac.uk/spm/). An 8196 vertex template cortical mesh was used. The forward model was computed using the EEG BEM (boundary element model) head model. The inverse reconstruction was computed using the multiple sparse priors (MSP) algorithm^45^. Source estimates for the two time windows of interest (130–166 ms and 234–290 ms; i.e., the time periods of the early and later IC-sensitive ERP components. See Fig. 4 in the Results section) were generated for the difference waveforms corresponding to the six contrasts. Note that SPM reconstructs changes in source activity, rather than activity per se, and thus it is recommended to limit the time window to the activity of interest^44^. Specifically, for the current data, if the entire epoch of −100 to +400 ms was used, the early IC-sensitive ERP component would be treated as error because its amplitude was smaller than that of the later IC-sensitive ERP component. Therefore, the source reconstruction was performed separately on the two time windows. MRIcron (www.mccauslandcenter.sc.edu/mricro/mricron) was used for the presentation of the results.

## Author Contributions

W.X. and Z.D.R. designed the research; W.X. performed the research; W.X. analyzed the data; Z.L., Q.C. and G.L.Y. made the figures; W.X. wrote the paper. All authors commented on and edited the manuscript. All authors approved the final version of the manuscript for submission. Affiliation 1 and 2 contributed equally to this work.

## Supplementary Material

Supplementary Information Supplementary Information
