# Supplementary material for: Attentional modulations of the early and later stages of the neural processing of visual completion
Source: Sci Rep. 2015 Feb 10;5:8346. doi: 10.1038/srep08346 (PMC4322362; doi:10.1038/srep08346)
Supplement: Supplementary Information [file srep08346-s1.pdf]

Title: Attentional modulations of the early and later stages of the neural processing of visual completion

Authors: Xiang Wu[1, 2 *], Liang Zhou[1], Cheng Qian[1], Lingyu Gan[1], Daren Zhang[2]

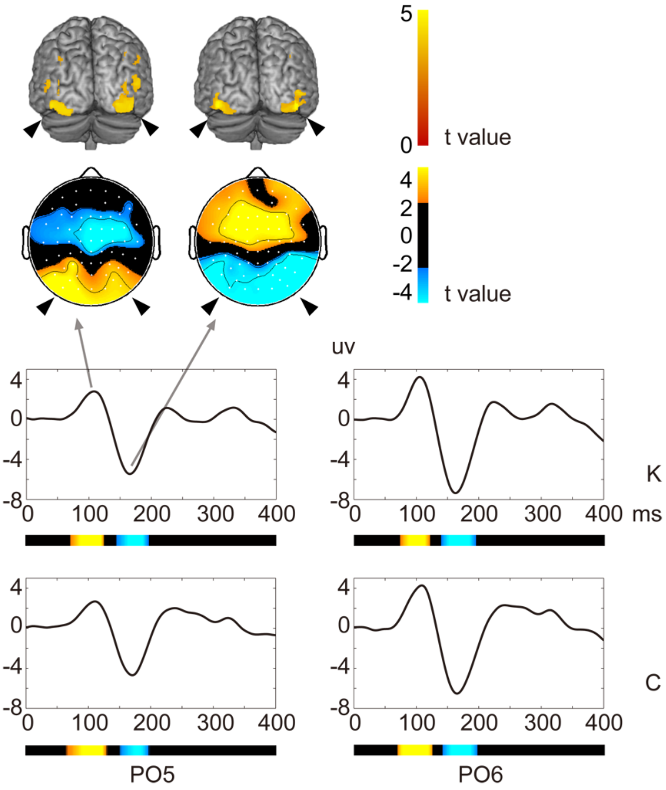

Fig. S1. Illustration of the P1 and N1. The ERP waveforms elicited by the Kanizsa (K) and control (C) stimuli in the KF task are shown. Below the waveform images are the statistical maps that show the time periods in which the waveforms were significantly different from 0 (black indicates non-significance differences). The typical VEP components P1 and N1 can be clearly observed. Statistical topographic maps for the P1 and N1 in response to the Kanizsa figure (indicated by the long transparent arrows; 70-124 ms after stimulus onset for the P1, and 148-196 ms for the

N1) are shown above the waveform images. The arrows indicate the scalp areas where the P1 and N1 occurred. Statistical maps from the statistical source analyses of the P1 and N1 are shown at the top (uncorrected $p<0.001$). The arrows indicate the strongest source activities underlying the P1 and N1.
